# Bellamy’s Guide to Surgical Anatomy

**Published:** 1874-04

**Authors:** 


*The Students Guide to Surgical Anatomy; Being a Description of the Most Important Surgical Regions of the Human Body, and intended as an Introduction to Operative Surgery.* By Edward Bellamy, F. R. C. S. With Illustrations. Philadelphia: Henry C. Lea, 1874. Buffalo: T. Butler & Son.

This book is intended to assist Students to put in practical application their knowledge of Anatomy. It contains in a condensed form some very valuable information, and as a guide to operative surgery, will be of much service. The subjects are for the most part well illustrated, many of the figures being original in this work. The book will be of value to students who wish to refresh their minds upon certain points in Surgical Anatomy, and who do not desire to search through the more extended treatises.

